# Drivers of wolf depredation reporting and compensation use intentions by livestock producers

**DOI:** 10.7717/peerj.20732

**Published:** 2026-02-02

**Authors:** Rae Nickerson, Rebecca M. Niemiec, Alexandra Few, Dana Hoag, Paul H. Evangelista, Stewart W. Breck

**Affiliations:** 1 Department of Ecosystem Science and Sustainability, Colorado State University, Fort Collins, CO, United States of America; 2 Department of Human Dimensions of Wildlife, Colorado State University, Fort Collins, CO, United States of America; 3 Jackson Hole Land Trust, Jackson, WY, United States of America; 4 Department of Agriculture and Resource Economics, Colorado State University, Fort Collins, CO, United States of America; 5 Wildlife Services, United States Department of Agriculture, Fort Collins, CO, United States of America

**Keywords:** Compensation programs, Theory of planned behavior, Human wildlife conflict, Psychological drivers, Behavioral intentions, Conflict reporting, Carnivore depredation, Wildlife management, Wolf-livestock, Human wildlife coexistence

## Abstract

**Background:**

Across the western United States, compensation programs that pay livestock producers for losses seek to mitigate the impact of carnivore depredation on livestock. However, data suggest not all livestock producers report wolf depredations or utilize compensation programs. Understanding factors influencing producers to report depredations and to use compensation programs will be critical to program efficacy.

**Methods:**

We designed a questionnaire expanding the Theory of Planned Behavior to explore which social-psychological and demographic factors most strongly correlate with compensation use and conflict reporting intentions. Our questionnaire was distributed across the inland western United States with confirmed wolf populations including Washington, Oregon, California, Idaho, Montana, Wyoming, Colorado, Arizona, and New Mexico, as well as Alberta, Canada.

**Results:**

Perceptions of how commonly other producers were reporting wolf depredation (descriptive norms) and worry about future wolf depredation (perceptions of risk probability) were the strongest predictors of reporting intention. A lack of trust in state agencies, perceived financial risk without access to depredation compensation, descriptive norms, attitudes, age, and producer state of residence were the strongest predictors of compensation use intention. Our findings suggest that reducing operational financial vulnerability, building trust in agency personnel, and peer-to-peer knowledge sharing may be effective strategies for increasing reporting and compensation use. Though our sample size was relatively small, our results are informative and have important implications for compensation programs and reporting.

## Introduction

Carnivore-livestock conflicts (hereafter, conflicts) are one of the most contentious aspects of large carnivore conservation and management (Van Eeden et al., 2020). Through a combination of natural expansion and two reintroduction efforts in the 1990’s, wolves (*Canis lupus*) have reestablished across the Western United States (DeCesare et al., 2018; Martin et al., 2025). The negative economic impacts of wolves are disproportionately borne by livestock producers, often in varied and unequal ways (DeCesare et al., 2018; Martin et al., 2025). Wolf-livestock conflict can result in direct economic losses to the producer in the form of depredation (when wolves kill livestock) and indirect losses from predator-induced stress, such as reduced weight gain, reduced reproductive rates, and injuries needing veterinary care (Steele et al., 2013; Ramler et al., 2014). In 2015, 280,600 head of cattle and calves were estimated to have been killed in the USA by predators at an estimated loss of $7.1 million caused by wolves (USDA, 2015). In another study published in 2014, cow-calf pairs grazed in areas where wolf depredation occurred lost an average of 22 pounds from indirect losses alone, resulting in an average loss of $6,679 per ranch (Ramler et al., 2014). In addition, there can be large time, energy, and resource investments required by livestock producers to monitor wolves and livestock and manage conflict coordination (*e.g.*, additional hours and employees, changing grazing protocols, investigating and reporting depredations; Dickman, Macdonald & Macdonald, 2011; Anderson et al., 2024). One study found that both carnivore attacks and high carnivore densities were associated with lower overall operational gains for sheep production in Sweden (Widman, Steen & Elofsson, 2019). Adapting to expanding predator populations on top of changing climate, ecological, and market conditions can force ranchers to sell their operations, thus risking the conversion of rangelands to development. This development can result in the loss of crucial ecosystem services that support local, state, regional, even global human and wildlife communities (Martin et al., 2025; Smith et al., 2025).

To mitigate the negative impacts of wolf depredation on livestock operations, several state and federal agencies, as well as some non-government organizations, offer compensation programs that pay producers for confirmed wolf depredations (Ravenelle & Nyhus, 2017). How these programs are administered and funded varies widely. Many programs offer a 1:1 fair market price for livestock killed, while some states have multipliers, or rarely pay-for-presence models (see Nickerson et al., 2024; Martin et al., 2025 for descriptions of various North American compensation programs). Despite their intended benefits to producers, not all producers see them positively, apply for compensation, or even report depredations (Nickerson et al., 2024).

Several studies have begun investigating the barriers to compensation use such as producer perceptions of program utility and structure (Montag, Patterson & Sutton, 2003), whether compensation improves tolerance for predators and/or fosters coexistence (Naughton-Treves, Grossberg & Treves, 2003), and whether compensation improves the economic equity and viability of ranching (Muhly & Musiani, 2009; Lee et al., 2017). However, no study has yet to systematically and quantitatively measure the social-psychological factors (outlined below) influencing whether producers use compensation. Measuring tolerance for wolves, defined by Harris (2020) as reduced predator killings, improved producer attitudes toward predators, and improved compliance with conservation protocols, is not the same as measuring motivators for compensation use, or compensation’s ability to improve the viability of ranching in the face of wolf conflict. Compensation programs may be important for the fair distribution of economic resources regardless of whether they build tolerance or foster coexistence (Dawson, Martin & Danielsen, 2018).

Additionally, the link between depredation reporting and compensation use is understudied. Compensation programs often require producers report suspected depredations and have them investigated and confirmed before they can apply for compensation (Steele et al., 2013), potentially linking the two behaviors. We know very little about what factors motivate or deter depredation reporting behavior, or how producer experiences with reporting may influence compensation use, or if experiences with compensation influence reporting behavior. Lee et al. (2017) found that the majority of Canadian livestock producers experiencing wolf and/or grizzly bear depredation did not report or apply for compensation due to reporting and to compensation-related processes and/or requirements. Additionally, voluntary livestock depredation reporting provides important information for local wildlife managers and neighboring producers about the frequency, location, and severity of conflicts with carnivores (Steele et al., 2013). Since reported depredations are used to develop conflict-related policy and management protocols, understanding whether factors related with either behavior are influencing reporting or compensation use may help wildlife managers and policy makers improve reporting rates, compensation utilization, and the overall effectiveness of management.

### The theory of planned behavior

Portions of this text were previously published as part of a thesis (Nickerson, 2021). Applying an expanded version of the Theory of Planned Behavior (TPB—Fishbein & Ajzen, 2011), we used an exploratory approach to examine the social-psychological factors associated with behavioral intentions related to wolf depredation reporting and compensation use. The TPB is a social-psychological framework stating that behavioral intentions are the strongest driver of behavior, and these intentions are driven by the three following social-psychological constructs: attitudes, injunctive norms, and perceived behavioral control. The attitude construct measures whether an individual finds a behavior favorable or not. Injunctive norms measure whether an individual believes they are expected to engage in a behavior by others they consider relevant, such as neighboring producers, the agricultural community, or friends and family. Perceived behavioral control measures whether an individual believes they have the ability to effectively engage in a behavior (Nigbur, Lyons & Uzzell, 2010). By modeling behavioral intention instead of behavior, the perspectives of producers who have yet to experience depredation can be included, which are also relevant to future policy and wolf management.

The Theory of Planned Behavior is widely accepted as a framework strongly predictive of behavior, including behaviors related to wildlife management and conservation (Van Eeden et al., 2020) and livestock production (Armitage & Conner, 2001; Borges et al., 2014). While no studies to our knowledge have specifically applied the TPB to compensation and reporting decisions, Wilbur et al. (2018) found a strong relationship between attitudes about black bear management decisions and bear conflict reporting behavior, and both qualitative and quantitative studies have found that norms motivate a variety of behaviors related to agriculture and natural resources, including the adoption of new cattle management techniques (Willcox, Giuliano & Monroe, 2012; Borges et al., 2014).

We expanded the TPB in multiple ways to build off prior literature. First, we added descriptive norms—perceptions of how commonly performed a behavior is Cialdini, Kallgren & Reno (1991), and personal norms—self-based standards of behavior an individual expects of themself, tied directly to one’s values and expectations of desired or undesired outcomes (Schwartz, 1973; Niemiec et al., 2020). Recent meta-analyses suggest these types of norms can influence pro-environmental behavior (Niemiec et al., 2020). Second, we expanded our model by adding metrics for perceived risk, beliefs about the utility or reporting and compensation programs, trust, and demographic factors, which previous research suggests may be important for understanding compensation use and reporting behavior (Wilbur et al., 2018; Lute et al., 2017; Slovic, 1987). Following the theory of Walpole & Wilson (2020) we added metrics for past experience with the risk (referred to as affective risk—depredation in our case), perceived severity of the risk event (financial vulnerability), and perceived probability/vulnerability of the risk (level of worry about potential wolf depredation). Additionally, we added belief metrics for each behaviors’ perceived utility, as is common in TPB literature examining the adoption of new techniques, programs, or protocols (Borges et al., 2014; Lute et al., 2017).

Trust has been identified as a significant driver of behaviors related to natural resources and agriculture (Davenport et al., 2007; Lute et al., 2017). Coleman & Stern (2017) define trust as “a psychological state in which an entity (a trustor) accepts some level of vulnerability based on a positive expectation of another entity (a trustee)” (p. 119). Both a lack of trust in management authorities/personnel, and in management decisions and processes have been found to influence reporting behavior specifically (Wilbur et al., 2018), and it is likely that different types of trust (*e.g.*, dispositional, procedural, affinitive, *etc*.) play different roles in decision making related to wolf depredation and compensation (see Coleman & Stern, 2017). Adapting these methods, we included trust questions for state and federal agencies, environmental groups, and the depredation confirmation process in our model (Coleman & Stern, 2017).

Finally, demographic factors are commonly used in studies examining human behavior and wildlife, although with varying degrees of predictive power (Hayman et al., 2014; Wilbur et al., 2018). We examined age, gender, state of residence, number and type of livestock owned and/or grazed, and whether respondents graze on public and/or private lands. The number of livestock may influence the perceived severity of a depredation event on the economics of an individual operation, and furthermore the intention to report or use compensation. Whether livestock are grazed on public or private lands, the type of livestock, and state of residence can influence livestock risk to wolf depredation, the amount of compensation available to the landowner, and the likelihood of depredation detection (Oakleaf, Mack & Murray, 2003), all of which may influence behavioral intentions.  

Due to the nature of compensation and reporting behaviors being linked, we chose to test this quantitatively, providing a novel, theoretical contribution. We did this by using our expanded TPB model to create four behavioral intention models—one model where only TPB factors associated with reporting behavior were included to model reporting intentions (specific reporting model), a second model where only TPB factors associated with compensation use behavior were included to model compensation use intentions (specific compensation model) and two mixed models where TPB factors related to both behaviors were included to predict reporting intentions (mixed reporting model) and compensation use intentions (mixed compensation model). Our research questions were: (1) which behavior-specific variables in the expanded TPB predict reporting behavioral intentions, (2) which behavior-specific variables in the expanded TPB predict compensation use behavioral intentions, and (3) do reporting-specific variables predict compensation use behavioral intentions, and vice versa?

## Materials and Methods

### Study area

Our study area included all inland western states in the US that had confirmed wolf populations including Washington, Oregon, California, Idaho, Montana, Wyoming, Colorado, Arizona, and New Mexico. We focused on these states because ranching primarily occurs on large tracts of open rangeland that is a mix of public and private ownership. Wolf predation can be more challenging to manage on these landscapes and thus compensation becomes a more important tool for managing conflict. Depending on livestock conflict history, wolf status, and other factors, compensation programs vary widely from state-to-state in their funding, administration, and associated reporting protocols (Nickerson et al., 2024). To successfully capture the diversity of experiences with reporting wolf depredation and compensation use, we targeted livestock producers in all nine-mainland western United States as well as Alberta, Canada. Alberta province is part of the Northern Rocky Mountain Range and shares a very similar socio-ecological system of wolf-livestock conflict to that of the western United States, including compensation programs (Lee et al., 2017).  Prior research has demonstrated that wolf habitat selection, connectivity and management are similar in both countries (Van den Bosch et al., 2023; Carroll, McRae & Brookes, 2011).

### Questionnaire design and implementation

Livestock producers, wildlife managers, researchers, and NGO staff reviewed a draft of the questionnaire after approval by the Colorado State University Institutional Review Board (CSU IRB–FWA A0000647–protocol #20-10064H). To distribute the questionnaire, we partnered with the non-profit organization Western Landowners Alliance (WLA), an organization with a mission to advance policies and practices that sustain working lands, connected landscapes, and native species. We used purposive sampling *via* snowballing to distribute our questionnaire, as these techniques allowed us to focus on, and more easily identify, livestock producers living in areas with wolves (Etikan, Musa & Alkassim, 2016). Using an anonymous link, we emailed the questionnaire to all Western Landowners Alliance’s over 1,000 landowner and land steward members (many of which are livestock producers) and other stakeholder members (hereafter constituents). Next, we emailed state and county-level Cattlemen’s, Wool, and Beef Growers’ Associations, State and Tribal Farm Bureaus, Extension agents from western universities, and agriculture and wildlife agency personnel to try to include the diversity of opinion leaders that producers seek information from regarding agricultural issues (Dillman, Smyth & Christian, 2014). After completing the questionnaire, participants were encouraged to share the questionnaire link with other livestock producers west-wide (Etikan, Musa & Alkassim, 2016). Our team sent two reminder emails in January and March of 2021, then closed the questionnaire in May 2021 with a total of *n* = 165 responses. Due to the prioritization of anonymity and our snowballing technique, we did not track how many producers received the questionnaire regardless of whether they took the questionnaire. Based on original email lists, we estimate that at least 1,100 people received the questionnaire from our team directly.

We launched the questionnaire in December 2020 *via* Qualtrics (Qualtrics, Provo, UT, USA), a licensed online questionnaire platform. Survey participants were informed by email with survey link that their participation was completely voluntary, that they may withdraw their consent and stop participation at any time by simply not providing answers to the survey questions, and that by clicking on the survey link, they were providing their consent. We structured the questionnaire based on a modified mixed-methods version of the Tailored Design Method (Dillman, Smyth & Christian, 2014). The questionnaire contained five sections: (1) Questions about operational characteristics, (2) questions about the reporting process and reporting intention (reporting related TPB variables found here), (3) questions about the compensation process and compensation intention (compensation related TPB variables found here), (4) questions about an ideal compensation program (see Nickerson et al., 2024), and (5) demographic and descriptive questions (see Tables S1 and S2 for all coding). We chose not to ask a personal norm question related to compensation use as it has almost exclusively operational/individual scale benefits. Aside from nine categorical questions, we asked all TPB constructs on a continuous five or seven-point Likert scale (Joshi et al., 2015). Questions using the “extremely positive” to “extremely negative”, “extremely worried” to “not at all worried”, and “extremely likely” to “not at all likely” Likert scales used a 5-point scale. Questions using the “strongly agree” to “strongly disagree” Likert scale used a 7-point scale.

### Analysis

As part of cleaning our data for analyses, we removed questionnaire responses with thirty percent or more questions unanswered, and/or responses without a response to the modeled dependent variable, reporting intention and compensation use intention, respectively (Wu et al., 2009). Unfortunately, this left us with no usable responses from Washington State or Alberta, Canada (although responses were still used for descriptive statistics). We first tested for correlation among continuous predictor variables across all four models, finding correlations of *r* = 0.7 or higher for only two variable pairs: (1) Between the reporting and compensation descriptive norm variables (*r* = 0.78), and (2) between the compensation related perceived behavioral control variables (*r* = 0.75). We chose to keep both the reporting and compensation descriptive norms since they addressed separate behaviors. Perceived behavioral control was operationalized as the average response across two survey items for each participant. The two items showed high reliability, with Cronbach’s *α* = 0.85 (Wu et al., 2009). Finally, we replaced any remaining missing values across individual question responses with the mean of the total sample (Wu et al., 2009). On each of our four models—the specific reporting and compensation use models, and the mixed reporting and compensation use models—we ran Least Absolute Shrinkage Selector Operator (Lasso) regression as a regularization method before running Ordinary Least Squares (OLS) regressions to identify significant variables for reporting and compensation use (Tibshirani, 1996; Wan, Datta & Conklin, 2015; McNeish, 2015; Niemiec et al., 2016; Mainzer et al., 2024). Finally, we made adjustments to *p*-values to account for multiple testing (Narum, 2006; Benjamini & Hochberg, 1995). As a sensitivity test, we ran all four models using multiple imputation *via* chained equations (MICE—Van Buuren & Groothuis-Oudshoorn, 2011) instead of mean replacement before running OLS on the Lasso selected variables (Wan, Datta & Conklin, 2015; Mainzer et al., 2024). This comparison allowed us to validify the Lasso regularization approach with mean replacement. Details of this analysis can be found in File S1.

## Results

### Description of the sample

Our questionnaire resulted in 130 usable responses for the specific reporting model and descriptive statistics, 127 for the mixed reporting model, and 128 responses for both compensation models (data for all four models had less than 17% unanswered questions—see Table 1 for general findings and Tables S1 and S2 for detailed descriptive statistics for the total surveyed population).

**Table 1:** Key findings from our analyses. Detailed results can be found in Tables 2 and 3, and survey details in Tables S1 and S2.

**Reporting**	• Eighty percent of respondents said they were likely or highly likely to report future wolf depredation.
	• Those with reporting experience were more likely to believe the reporting process to be time consuming, useful, and depredation investigations done accurately.
	• The higher the percentage of neighbors and/or community members a respondent believed were already reporting/would report depredation, the more likely their intention to report.
	• The more worried a participant was about wolf depredation, the more likely they were to intend to report.
**Compensation**	• Eighty percent of respondents said they were likely or highly likely to apply for wolf depredation compensation in the future.
	• Sixty-nine percent of those who had experienced wolf depredation in the past had applied for compensation at least once.
	• Those with compensation experience were less likely to believe the compensation process to be difficult or time consuming.
	• A lack of trust in state agencies made someone less likely to intend to apply for compensation.
	• The higher percentage of neighbors and/or community members a respondent believed were already using depredation compensation/would apply for compensation, the stronger their intention to use compensation.
	• The more financially vulnerable a respondent perceived to be to wolf depredation, the more likely their intention to use compensation.
	• The more positive a respondent’s attitude toward being compensated for depredation, the higher their intention.
	• Respondents from Colorado, New Mexico, and Montana were more likely to intend to use compensation.
	• The older a respondent, the less likely their intention to use compensation.
	• Seventy-four percent of respondents believed the compensation available to them was not representative of their actual losses to wolves.

Although 80% of respondents (*n* = 104) said they would be likely to highly likely (likely from here forward) to report future wolf depredation, 23% of respondents with reporting experience had chosen to not report a depredation at some time (*n* = 11). In general, those with reporting experience (*n* = 48—“reporters” from here forward) believed the reporting process to be more time consuming than those who had never reported a depredation (3% more) and believed reporting to be slightly more useful (13% more). Trust in the accuracy of depredation confirmations was 29% higher among the reporters and reporters were 13% more likely to intend to report future depredation than non-reporters. All three reporting related norms were higher among reporters (90% agree for injunctive, 92% for descriptive, and 83% for personal).

Of the total respondents, 80% (*n* = 104) stated they would be likely to apply for depredation compensation in the future. Of the producers who had experienced wolf depredation (*n* = 55—“depredation population” from here forward), 69% had applied for compensation at least once. Of the total compensation users (*n* = 38—“compensation users” from here forward), 65% stated to have a positive or extremely positive general attitude toward being compensated. Seventy-four percent of compensation users believed the compensation available to them was not representative of their actual losses to wolves. Compared to the total surveyed population, 8% less of compensation users agreed that the application process was difficult or time consuming. Both injunctive and descriptive norms were higher among compensation users, (82% for both norms) and they were 18% more likely to intend to use compensation in the future than the total surveyed population.

Trust that the agency personnel investigating depredations were doing so fairly was about 30% higher among reporters and compensation users than the total surveyed population. Perceived risk probability/vulnerability was 16% higher among reporters than the total surveyed population, but perceived risk severity was 10% lower. Perceived risk probability/vulnerability and perceived severity were almost identical among reporters and compensation users.

### Significant predictors of reporting intention

The strongest predictor of reporting intention within our surveyed population for both the mixed and specific reporting models was our descriptive norm (see Table 2). The higher the percentage of neighbors and/or community members a respondent believed were already reporting/would report depredation the more likely their intention to report in the future. Perceived probability of risk was only significant in the specific reporting model, meaning the more worried a participant was about wolf depredation, the more likely they were to intend to report. Our missingness indicator variable for past experience with depredation/risk created during our mean-replacement process was significant in the mean replacement mixed reporting model, and our missingness indicator variable for age was significant in both the mixed and specific reporting models—meaning that data may not have been missing at random (Little & Rubin, 2019).

**Table 2:** Results from Lasso regressions—one using mean replacement (Mean) and the other using multiple imputation (MI)—predicting reporting intention (mixed and specific).

**Variable**	**Mean** **BC**	**Mean** ***p*-value**	**Mean SE**	**Mean** **BH**	**MI** **BC**	**MI** ***p*-value**	**MI** **SE**	**MI** **BH**
**Reporting Mixed Model:** Mean Adjusted R-squared: 0.38 Mean Model *p*-value: < 0.001								
Raise sheep	−0.139				−0.129			
Perceived probability risk	0.105				0.161			
Attitude reporting	0.043				0.074			
**Reporting** **descriptive norm**	**0**.**287**	***p* < 0.01**	**0.087**	***p* < 0.05**	**0**.**224**	***p* < 0.05**	**0.090**	**not sig**
Reporting utility belief	0.145				0.162			
Reporting personal norm	0.136				0.154			
**Age - missing**	**0**.**212**	***p* < 0.01**	**0.327**	***p* < 0.05**	NA	NA	NA	NA
Trust management/process	0.106				0.151			
Reporting injunctive norm	0.030				0.078			
**Past experience risk –** **missing**	**−0**.**146**	***p* < 0.05**	**0.323**	**not sig**	NA	NA	NA	NA
Attitude compensation	0.067				0.013			
Perceived risk severity	−0.132				0.066			
Attitude reporting –missing	−0.101				NA			
Age –MI model only	NA				−0.022			
Past experience risk –MI Model only	NA				−0.148			
**Reporting Specific Model:** Mean Adjusted R-squared: 0.36 Mean Model *p*-value: <0.001								
Raise sheep	−0.135				−0.123			
**Perceived probability risk**	**0**.**184**	***p* < 0.05**	**0.057**	***p*= 0.05**	**0**.**216**	***p* < 0.01**	**0.062**	**not sig**
Attitude reporting	0.070				0.063			
**Reporting** **descriptive norm**	**0**.**287**	***p* < 0.01**	**0.087**	***p* < 0.05**	**0**.**233**	***p* < 0.01**	**0.089**	**not sig**
Reporting utility belief	0.146				0.157			
Reporting personal norm	0.143				0.154			
**Age - missing**	**0**.**200**	***p* < 0.01**	**0.281**	***p* < 0.05**	NA	NA	NA	NA
Trust management/process	0.089				0.154			
Reporting injunctive norm	0.037				0.085			
Past experience risk – missing	−0.144				NA			
Attitude reporting –missing	−0.100				NA			
Age –MI model only	NA				−0.036			
Past experience risk –MI Model only	NA				−0.164			
Perceived risk severity –MI model only	NA				−0.001			

**Notes.**

Lasso-selected predictors are listed with beta coefficients (BC), standard errors (SE) and *p*-values both before and after Benjamin-Hochberg adjustments (BH−alpha = 0.05). Significant variables from the mean replacement regression are bolded and listed on the left (Mean), and significant variables from the multiple imputation regression are bolded and listed on the right (MI). *P*-values that differ between regressions are underlined. “not sig” means not significant.

Using multiple imputation instead of mean-replacement, only the reporting descriptive norm was significant in the mixed reporting model. Missingness indicators were not included in the multiple imputation models since they were a product of the mean-replacement process only. Both the reporting descriptive norm and perceived probability of risk variables remained significant in the specific model. Perceived probability of risk became slightly more significant in the specific multiple imputation model than in the specific mean-replacement model. No compensation-related variables were significant in any of the four reporting-related models.

### Significant predictors of compensation use intention

Based on our modeling effort, trust, perceived risk severity, descriptive norms, attitudes, age, and state of residence had the strongest relationship with compensation use intention (see Table 3). In both the mixed and specific compensation models, a lack of trust in state agencies made someone less likely to intend to apply for compensation. The higher percentage of neighbors and/or community members a respondent believed were already using depredation compensation/would apply for compensation, the stronger their intention to use compensation. The more financially vulnerable a respondent perceived to be to wolf depredation, the more likely their intention to use compensation in both the specific and mixed models. The more positive a respondent’s attitude toward being compensated for depredation, the higher their intention. Respondents from Colorado, New Mexico, and Montana were more likely to intend to use compensation in the mixed model, but not Montana residents in the specific model. The older a respondent, the less likely their intention to use compensation in the mixed model, but age was not significant in the specific model.

**Table 3:** Results from Lasso regressions—one using mean replacement (Mean) and the other using multiple imputation (MI)—predicting compensation use intention (mixed and specific).

**Variable**	**Mean** **BC**	**Mean** ***p*-value**	**Mean SE**	**Mean** **BH**	**MI** **BC**	**MI** ***p*-value**	**MI** **SE**	**MI** **BH**
**Compensation Use Mixed Model:** Mean Adjusted R-squared: 0.45 Mean Model *p*-value: < 0.001								
California resident	0.253				0.259			
Idaho resident	0.041				0.061			
**Trust state government**	**−0**.**218**	***p* < 0.01**	**0.051**	***p* < 0.05**	**−0**.**243**	***p* < 0.01**	**0.051**	***p* < 0.05**
**Compensation** **descriptive norm**	**0**.**277**	***p* < 0.01**	**0.089**	***p* < 0.05**	**0**.**257**	***p* < 0.01**	**0.089**	***p* < 0.05**
Oregon resident	0.205				0.204			
**Colorado resident**	**0**.**468**	***p* < 0.01**	**0.512**	***p* < 0.05**	**0**.**494**	***p* < 0.01**	**0.517**	***p* < 0.05**
**New Mexico resident**	**0**.**326**	***p* < 0.05**	**0.540**	**not sig**	**0**.**325**	***p* < 0.05**	**0.548**	***p* < 0.05**
Wyoming resident	0.086				0.105			
Perceived probability risk	0.065				0.061			
Trust management/process	0.022				0.081			
Reporting injunctive norm	0.083				0.056			
**Attitude compensation**	**0**.**183**	***p* < 0.05**	**0.089**	**not sig**	**0**.**232**	***p* < 0.01**	**0.096**	***p* < 0.05**
**Montana resident**	**0**.**426**	***p* < 0.05**	**0.487**	**not sig**	**0**.**450**	***p* < 0.05**	**0.497**	**not sig**
Compensation injunctive norm	−0.021				0.007			
**Age**	**−0**.**153**	***p* < 0.05**	**0.118**	**not sig**	**−0**.**139**	**not sig**		**not sig**
**Perceived risk severity**	**0**.**230**	***p* < 0.01**	**0.059**	***p* < 0.05**	**0**.**230**	***p* < 0.01**	**0.060**	***p* < 0.05**
Reporting utility belief	0.104				0.109			
Attitude reporting- missing	0.108				NA			
Reporting personal norm	0.166				0.153			
Attitude reporting –MI Model only	NA				−0.131			
**Compensation Use Specific Model:** Mean Adjusted R-squared: 0.42 Mean Model *p*-value: < 0.001								
California resident	0.228				0.226			
Idaho resident	0.040				0.037			
**Trust state government**	**−0**.**380**	***p* < 0.001**	**0.065**	***p* < 0.01**	**−0**.**382**	***p* < 0.001**	**0.065**	***p*< 0.01**
**Compensation** **descriptive norm**	**0**.**220**		**0.091**	***p*= 0.05**	**0**.**218**	***p* < 0.05**	**0.091**	**not sig**
Oregon resident	0.164	***p* < 0.05**			0.161			
**Colorado resident**	**0**.**451**		**0.520**	***p*= 0.05**	**0**.**449**	***p* < 0.01**	**0.526**	***p*= 0.05**
**New Mexico resident**	**0**.**298**	***p* < 0.01**	**0.549**	**not sig**	**0**.**293**	***p* < 0.05**	**0.552**	**not sig**
Wyoming resident	0.068	***p* < 0.05**			0.066			
Perceived probability risk	0.081				0.078			
Trust management/process	0.092				0.093			
Trust federal government	−0.173				−0.173			
**Attitude compensation**	**0**.**209**		**0.090**	***p*= 0.05**	**0**.**212**	***p* < 0.01**	**0.091**	***p*= 0.05**
**Montana resident**	**0**.**389**	***p* < 0.05**			0.379			
Compensation injunctive norm	0.065				0.063			
**Age**	−0.134				−0.130			
**Perceived risk severity**	**0**.**210**	***p* < 0.05**	**0.060**	***p*= 0.05**	**0**.**207**	***p* < 0.05**	**0.060**	**not sig**

**Notes.**

Lasso-selected predictors are listed with beta coefficients (BC), standard errors (SE) and *p*-values both before and after Benjamin-Hochberg adjustments (BH−alpha = 0.05). Significant variables from the mean replacement regression are bolded and listed on the left (Mean), and significant variables from the multiple imputation regression are bolded and listed on the right (MI). *P*-values that differ between regressions are underlined. “not sig” means not significant.

Using multiple imputation instead of mean-replacement, all significant predictors from the mean-replacement models remained significant, except for age in the mixed model. Attitude compensation became slightly more significant in the mixed model (see Table 3). No reporting-related variables were significant in either the mixed of specific compensation models.

## Discussion

Our intention with this study was to gain a better understanding of factors that drive intentions of wolf depredation reporting and compensation use in the western US and Alberta, Canada by adapting the Theory of Planned Behavior for an exploratory approach. Though our sample size was relatively small, our results are informative and have important implications for compensation programs. Our findings highlight the potential importance of descriptive norms, perceived risk, and trust as motivators of reporting and compensation use intentions. Although several Lasso-selected predictors lost slight significance when data was treated with multiple imputation instead of mean replacement as a sensitivity test to the Lasso approach, only one predictor—age in the mixed compensation model—did not remain significant across both approaches. This confirmed that our mean-replaced significant predictors were robust to the imputation approach, supporting the stability of their significance (Wan, Datta & Conklin, 2015; Mainzer et al., 2024). We also explored interactions between reporting and compensation use intentions and found that no factors related to compensation drove reporting intention, and no factors related to reporting drove compensation use intention. These findings contribute novel insight to what aspects of compensation use and depredation reporting may drive behavioral intentions.

Descriptive norms were significant in all four models, suggesting that perceptions about whether other producers are reporting and/or applying for compensation may drive behavioral intentions, and furthermore whether a producer chooses to report a depredation or apply for compensation. Although injunctive norms—whether or not an individual believed their community and/or neighbors would support them reporting and/or applying for compensation—and the reporting personal norm—the higher an individual’s expectations of themselves to report depredations so as to maintain an accurate record of wolf conflict were not significant predictors in any of the models, they were originally selected by the Lasso regressions in all models. These findings support the conclusions of Stauder (2023) and particularly Niemiec et al. (2020), who found personal and descriptive norms had a larger relative influence on conservation-related behavioral intentions than injunctive norms. These results suggest future research should include metrics for all three types of norms, and that increasing producer awareness about reporting and compensation use by other producers may most effectively increase rates of both wolf depredation reporting and compensation use.

If norms are driving behavioral intentions across the total producer population and not just our surveyed population, then efforts to increase reporting and compensation use should focus on building supportive norms. In their meta-analysis on social influence in the natural resources, Abrahamse & Steg (2013) argue that community volunteers who help inform other community members on certain issues (known as the block leader approach) are particularly effective at spreading information because of existing social networks, face-to-face interaction, and existing trust. Similarly, increasing visibility of a behavior can also build descriptive norms (Niemiec et al. 2021). However, Niemiec et al. (2021) warn that visibility efforts can backfire and lead to a defensive response if the targeted community interprets visibility efforts as negatively impacting their autonomy, or if messaging is too general, and not directed to a specific community.

Like the findings of Van Eeden et al. (2020), norms and perceived risk constructs were among the strongest drivers of compensation use and reporting behavioral intentions. This may suggest that producers are not making decisions based on affective heuristics, instead relying on norms, trust, and perceived risk severity, aligning with findings from other studies on potentially dangerous wildlife (Hayman et al., 2014; Wilbur et al., 2018; Van Eeden et al., 2020). Risk literature has long argued that as perceived control over a hazard increases, perceptions of risk will decrease (Slovic, 1987; Kahan et al., 2007). Producers who don’t perceive any benefit gained from predator presence may feel a lack of autonomy and control over predator-livestock conflict and carnivore conservation policies (Dickman, Macdonald & Macdonald, 2011). As we did not operationalize perceived benefit gained from wolf presence in our survey, future research should consider exploring the relationship between perceived benefit and depredation reporting and compensation use intentions. Naugle et al. (2020) suggest that wildlife managers and policy makers should consider co-producing policies and management strategies with livestock producers to ensure those policies and strategies serve landowner needs. Nickerson et al. (2024) found that producers facing wolf conflict were interested in programs that provided multiple options for financial, resource, and technical support related to wolf depredation rather than only an ex-post compensation option.

Although we did find that a lack of trust in state agencies made someone less likely to intend to apply for compensation, we did not find a direct relationship between trust in the management processes (specifically the depredation investigation process) and reporting or compensation use intentions. This was supported by our finding that normative beliefs were stronger drivers of reporting intention than this type of trust, which may mean that even without trust in management processes and personnel, producers report depredations because they believe they should, and because they believe that other producers are also reporting. Trust in state agencies is likely a perception combined of what Coleman & Stern (2017) call affinitive trust—trust stemming from an affinity for the agency, and procedural trust—trust grounded in approval/agreement in the monitoring and management decisions of the agency. Our finding may suggest that those with existing trust are more likely to apply for compensation, or that the process of investigating a depredation may build trust with the agency. Davenport et al. (2007) argue that increasing interpersonal trust between community members and agency personnel may improve affinitive and procedural trust in the agency overall. The authors recommend that wildlife agencies may want to increase the level of interaction between their personnel and the ranching community as a way to indirectly improve trust in the agency as a whole by hiring and/or contracting members of the local community, using local businesses, or providing trainings to personnel in local norms, values, and knowledge (Davenport et al., 2007). Whether these techniques can be applied to compensation use and reporting behaviors related to wolf depredation is unknown.

Reporting and compensation use intentions were high among both those with compensation experience at over 90% for both behaviors and the total surveyed population at around 80% for both behaviors, despite mixed responses to questions asking respondents to reflect on their experience with both behaviors. This could suggest that compensation for direct losses is valued among our surveyed producers facing wolf conflict, even when administered through a program perceived to be less than satisfactory. Nickerson et al. (2024) found that livestock producers facing wolf conflict preferred programs that provided some amount of depredation compensation over programs with no depredation compensation option.

State of residence was only significant for the compensation models, where respondents from Montana, New Mexico, and Colorado were more likely to intend to apply for compensation. After the recent state-mandated wolf reintroduction (Niemiec et al., 2021), Colorado state producers were likely concerned about future wolf-conflict and related financial vulnerability, which may have contributed to a desire for readily available financial assistance. Additionally, resource managers in Montana and New Mexico have been managing wolves for longer than states like Washington, Oregon, and California. Greater familiarity with wolves and their management might have given producers time to familiarize themselves with compensation in these states, improving reported compensation use intentions. Compensation programs also vary significantly from state to state regarding the amount of compensation available, the ease of applying for compensation, and which agency a producer must coordinate with to receive compensation, potentially influencing compensation use intention (Nickerson et al., 2024; Martin et al., 2025).

Attitudes about compensation were significant in all compensation-related models, meaning respondents with more positive attitudes about compensation were more likely to intend to apply. This supports the outcomes measured by Wilbur et al. (2018), who found that attitudes of tolerance towards bears (positive attitude) was linked to an increased likelihood to not report bear conflict in their survey population. In our case, a positive attitude towards compensation resulted in a higher likelihood of future compensation use intention. This may mean that improving attitudes about compensation application such as process and transparency, and about compensation features such as amount or timeliness may improve compensation use.

Our results indicate that the age of respondents and whether they had experienced a wolf depredation in the past were partially significant (Tables 2 and 3). Specifically, those not willing to report their age were more likely to intend to report wolf depredation, while those not willing to report whether they had experienced a wolf depredation in the past were less likely to intend to report depredation. This may reflect underlying differences between participants who declined to provide either their age or whether they had experienced wolf depredation in the past, and those willing to answer both questions. In this case, missing responses are not missing at random but rather reflect a subgroup with similar factors influencing their behavioral intentions (Little & Rubin, 2019). While both questions are sensitive in nature, it makes logical sense that someone unwilling to report their wolf depredation experience on a survey may be untrusting of both researchers and the government, and therefore also not interested in reporting depredation to the responsible agency (Davenport et al., 2007).

From a management perspective, it is favorable that compensation related variables are not influencing reporting rates, since high reporting rates lead to better data that can be used to help create more effective wolf management policies and provide a more accurate picture of actual conflict. It’s possible that within our surveyed population, the community-scale benefits associated with reporting are overpowering any potential correlation between reporting and compensation factors, as reporting can help neighboring operations by informing local wolf management, and operations region-wide by informing effective wolf policy. Given this finding, our surveyed producers may have a sense of personal responsibility to help keep accurate and up-to-date records of wolf conflict so that policies developed using reporting data will reflect their actual wolf conflict. Alternatively, the reporting process itself could be perceived by our participants as less difficult, time consuming, or labor intensive when compared to applying for compensation, and therefore worth the effort regardless of factors related to the compensation process. That no reporting related variables were found to be significant in either compensation model may mean that having access to compensation for wolf depredation is important to livestock producers, regardless of their experiences/opinions on reporting wolf depredation (Nickerson et al., 2024).

The results from our exploratory analysis were limited by several factors. Originally, we intended to distribute questionnaires by hand at several events for livestock producers. Due to Covid restrictions, we moved the questionnaire to a fully online format. Additionally, our snowball method for collecting questionnaire responses may have biased results. Since protecting anonymity was more important than tracking those who received the questionnaire, we were unable to determine a response rate. It is possible that WLA constituents were more likely to respond to the questionnaire than other livestock producers due to their existing engagement on landowner concerns. If our sample is biased toward WLA constituents, it could mean that certain statistics like our descriptive statistics are not accurately representative of the total producer population. For example, WLA constituents may be more likely to intend to report a depredation than non-constituents, or those who wouldn’t report and/or use compensation may not have chosen to take the questionnaire after receiving it. WLA constituents may have also had higher levels of trust in agencies due to their existing engagement. Future research should include in-person questionnaire deployment as we believe the trust built through initial interactions would have improved response rates, increased our sample size, and reduced our question missingness (Little & Rubin, 2019). Additionally, although purposive sampling and snowballing allowed us to more easily access our subpopulation of livestock producers operating on landscapes with wolves, a truly random sample or stratified random sample would also benefit the representational power of findings, allowing for inferential instead of exploratory analyses. A subsequent study with pre-planned hypotheses should be conducted to confirm the associations found in our study.

## Conclusion

Compensation programs that reimburse livestock producers for losses incurred by large carnivores are an important component not only for maintaining the wellbeing of livestock producers but also for maintaining carnivore restoration efforts and the success of long-term management (Muhly & Musiani, 2009; Lee et al., 2017; Naughton-Treves, Grossberg & Treves, 2003). Additionally, voluntary livestock depredation reporting provides important information for local wildlife managers and neighboring producers about the frequency, location, and severity of carnivore conflict (Steele et al., 2013). When losses go unreported, records underestimate the true extent of conflict, making it difficult to counter the refrain that wolves have insignificant impacts on livestock operations. Underestimated conflict can also weaken the policy justification for continued compensation funding and nonlethal tool support (Nickerson et al., 2024). Accurate reporting helps to justify resource allocation for producers like depredation compensation and helps wildlife managers adapt conflict minimization efforts to real conditions. Our findings suggest that while reporting and compensation use intentions were relatively high among our surveyed population (80%), understanding the key drivers of both intentions can inform the improvement of these policies and programs, in turn increasing reporting rates and compensation use. In particular, our results suggest that reducing operational financial vulnerability for livestock producers, building trust in agency personnel, and peer-to-peer knowledge sharing that increases visibility within the communities facing conflict may be effective strategies for increasing reporting and compensation use intention.

However, future studies not limited to online snowball sampling should aim to be hypothesis driven with more representative sampling and in-person engagement to validate and expand upon our findings. Future research directions may also include exploring the potential connections between reporting and compensation use in more detail, how the specifics of each state’s compensation program influences reporting and compensation use, testing whether community-led visibility strategies such as trusted rancher ambassadors or locally tailored outreach effectively increase reporting and compensation use behavior, and examining how trust building approaches, such as increased agency engagement with local communities, affect participation. Overall, this study helps to advance our understanding of the social-psychological dynamics that shape reporting and compensation behavioral intentions among livestock producers, with important implications for both wolf management and the design of human-wildlife coexistence programs.

##  Supplemental Information

10.7717/peerj.20732/supp-1 Supplemental Information 1 Depredation Reporting and Compensation Survey Regressions, Rae Nickerson, 2025, CCC Funding, CSU

10.7717/peerj.20732/supp-2 Supplemental Information 2 Compensation specific model final dataset

10.7717/peerj.20732/supp-3 Supplemental Information 3 Reporting specific model final dataset

10.7717/peerj.20732/supp-4 Supplemental Information 4 Reporting mixed model final dataset

10.7717/peerj.20732/supp-5 Supplemental Information 5 Compensation mixed model final dataset

10.7717/peerj.20732/supp-6 Supplemental Information 6 Qualtrics survey

10.7717/peerj.20732/supp-7 Supplemental Information 7 Details on statistical analyses methods

10.7717/peerj.20732/supp-8 Supplemental Information 8 Survey Codebook

10.7717/peerj.20732/supp-9 Supplemental Information 9 Survey constructs and questions used to measure each construct wolf depredation reporting and compensation use intentions survey N = 130 respondents (unless otherwise specified with **) with percentage of the sample for each survey question.

10.7717/peerj.20732/supp-10 Supplemental Information 10 Survey constructs and questions descriptive results wolf depredation reporting and compensation use intentions survey N = 130 total respondents (unless otherwise specified with **) with percentage of the sample for each survey question.

## Additional Information and Declarations

### Data Availability

The following information was supplied regarding data availability: The raw data and code are available in the Supplemental Files.
