# Supplementary material for: Drivers of wolf depredation reporting and compensation use intentions by livestock producers
Source: PeerJ. 2026 Feb 2;14:e20732. doi: 10.7717/peerj.20732 (PMC12875219; doi:10.7717/peerj.20732)
Supplement: Supplemental Information 6 [file peerj-14-20732-s006.pdf]

Filter Questions

Wolf-Livestock Depredation and Compensation Survey:

My name is Rachael, (Rae) Nickerson, and I am a graduate student at Colorado State University (CSU) in the Ecosystem Science and Sustainability Department. In collaboration with Alex Few from Western Landowners Alliance - an organization with a mission to advance policies and practices that sustain working lands, connected landscapes and native species - our research team is conducting surveys and interviews with landowners and producers across the West to better understand the real impacts of wolves on livestock. The goal of our research is to address a fundamental concern of the livestock community that the impacts of wolves on livestock are underestimated, and therefore available depredation compensation is not representative of the lived experiences of landowners operating on landscapes with wolves, (please see the summary below for more information).

We would like you to take this survey to help our team better understand wolf depredations from the landowner's perspective. Your participation will help us identify what would constitute a fair compensation program, and your responses are critical as we strive to bring local knowledge to the larger conversation surrounding wolves in the West. This study will take place in the following states from June 2020 - September 2021: Washington, Oregon, California, Idaho, Wyoming, Montana, Colorado, New Mexico, and Arizona. We hope to collect around 500 survey responses and 50 interviews from participants over the course of this study.

Western Landowners Alliance will offer 50 issues of their 'On Land Magazine' for free to the first 50 survey participants, and all survey participants will be offered a chance to win a 2-year subscription to the 'On Land Magazine' (4 issues total) via a raffle. 5 Participants out of all survey participants will win the raffle and receive a 2-year subscription.

Participation will take approximately 30 minutes. Your participation in this research is voluntary. If you decide to participate in the study, you may withdraw your consent and stop participation at any time by simply not providing answers to the survey questions. While there are no direct benefits to you, we hope to gain more knowledge on how depredation compensation and wolf management can better serve landowners.

This survey will ask about your experience with wolf depredations, reporting wolf depredations, and compensation for wolf depredations. It's important that you answer all questions in the order they appear, as the survey will provide you with specific questions depending on your previous responses.

The following questions will ask about your operation. All responses will remain anonymous.

Do you raise or manage any of the following livestock: cattle, sheep, or goats?

- ○ Yes
- ○ No

Please select which livestock you raise. (select all that apply)

- ☐ Cattle
- ☐ Sheep
- ☐ Goats
- ☐ ________ Other

Roughly how many head (of any age and sex class) do you manage in a typical year?

- ○ 500 head or Less
- ○ Between 500 and 1,000 head
- ○ Between 1,000 and 3,000 head
- ○ 3,000 head or more

On what lands do you typically graze livestock? (select all that apply)

- ☐ Public
- ☐ Private
- ☐ ________ Other

What state do you live in?

- Arizona
- California
- Idaho
- Montana
- New Mexico
- Oregon
- Washington
- Wyoming
- [ ] Other

What county in your state?

[ ]

Depredation Questions

The following section will ask about your experience with wolf depredations and reporting them. We believe understanding why someone may choose to report, or not report a wolf depredation is important to understanding whether depredation reporting protocols and compensation programs are working for landowners. All responses will remain anonymous.

Have you ever experienced a wolf depredation on your livestock or the livestock you manage?

- Yes
- No
- I'm not sure

About how many wolf depredations have you had in the last 3 years?

Have you ever reported a wolf depredation?

- Yes
- No
- I'm not sure

Why did you report the wolf depredation(s)?

When you report a wolf depredation, who do you typically report to? (select all that apply)

- State wildlife agency
- U.S. Fish and Wildlife Service
- Wildlife Services
- Local Extension agent
- County personnel or the Sheriff's office
- U.S. Department of Agriculture through their annual survey
- Tribal authorities
- Verification by an independent party
- Neighbor(s)
- I'm not sure
- ________ Other

Some landowners may choose to not report wolf depredations for a variety of reasons.

Was there a time when you chose NOT to report a wolf depredation?

- Yes
- No
- I'm not sure

Why didn't you report the wolf depredation(s)?

If you experience wolf depredation in the future, how likely are you to report the depredation(s)?

- Extremely likely
- Likely
- Neither likely or unlikely
- Unlikely
- Extremely unlikely

Do you receive assistance for nonlethal tools for wolf-related livestock conflicts, (financial or material)?

- Yes
- No
- I'm not sure

Please list who supplies the funding and/or materials

Typically, how worried are you about wolf depredations on your livestock?

- Extremely worried
- Moderately worried
- Somewhat worried
- Slightly worried
- Not at all worried

What number or wolf depredations per 100 head do you consider to be an unacceptable outcome of operating alongside wolves, (head of any age or sex class)?

Please select any of the following you consider to be part of your economic losses from operating with wolves on the landscape, (select all that apply).

- The fair market value of depredated livestock
- The fair market value of livestock depredated but never found
- Veterinary costs associated with wounded animals, (indirect losses)
- Weight loss in surviving animals due to stress, (indirect losses)
- Lower reproductive rates and/or higher abortion rates in surviving animals due to stress, (indirect losses)

The following question will ask for your opinion on direct and indirect losses. Direct losses are defined as the loss in fair market value for the depredated animal. Indirect losses are defined as the veterinary costs for surviving animals wounded by wolves, weight loss in surviving animals due to the stress of wolf presence, and lowered reproductive rates and/or increased abortion rates in surviving animals due to the stress of wolf presence.

Which one of the following best describes how you feel about direct and indirect losses associated with wolf presence?

- Direct losses are more financially damaging than indirect losses
- Direct losses are less financially damaging than indirect losses
- Direct and indirect losses are equally financially damaging
- I'm not sure

TPB Reporting

The following section will ask for your opinion regarding the process of reporting wolf depredations. The reporting process includes finding carcasses, having them confirmed as wolf kills, and reporting confirmed depredations to the required personnel. All responses will remain anonymous.

Would you say your general attitude towards reporting wolf depredations to the required personnel is positive, negative, or neutral?

- Extremely positive
- Positive
- Neither positive or negative
- Negative
- Extremely negative

Please indicate to what extent you agree or disagree with the following statements:

Reporting wolf depredations helps wildlife management agencies identify depredating wolves.

- Strongly agree
- Agree
- Somewhat agree
- Neither agree or disagree
- Somewhat disagree
- Disagree
- Strongly disagree

Reporting is important for maintaining an accurate record of wolf depredation.

- Strongly agree
- Agree
- Somewhat agree
- Neither agree or disagree
- Somewhat disagree
- Disagree
- Strongly disagree

I trust the personnel investigating a wolf depredation to investigate fairly.

- Strongly agree
- Agree
- Somewhat agree
- Neither agree or disagree
- Somewhat disagree
- Disagree
- Strongly disagree

I don't want the federal government involved in my operations.

- Strongly agree
- Agree
- Somewhat agree
- Neither agree or disagree
- Somewhat disagree
- Disagree
- Strongly disagree

I don't want the state government involved in my operations.

- Strongly agree
- Agree
- Somewhat agree
- Neither agree or disagree
- Somewhat disagree
- Disagree
- Strongly disagree

I don't want environmental groups involved in my operations.

- Strongly agree
- Agree
- Somewhat agree
- Neither agree or disagree
- Somewhat disagree
- Disagree
- Strongly disagree

Detecting carcasses depredated by wolves is time consuming.

- Strongly agree
- Agree
- Somewhat agree
- Neither agree or disagree
- Somewhat disagree
- Disagree
- Strongly disagree

Having carcasses confirmed by the required personnel as wolf depredations is time consuming.

- Strongly agree
- Agree
- Somewhat agree
- Neither agree or disagree
- Somewhat disagree
- Disagree
- Strongly disagree

I know who to call to report wolf depredations.

- Strongly agree
- Agree
- Somewhat agree
- Neither agree or disagree
- Somewhat disagree
- Disagree
- Strongly disagree

My neighbors and/or community would approve of me reporting wolf depredations to the required personnel.

- Strongly agree
- Agree
- Somewhat agree
- Neither agree or disagree
- Somewhat disagree
- Disagree
- Strongly disagree

What percentage of your neighbors and/or community that experience, (or might experience) wolf depredations do you think report, (or would report) those depredation(s)?

- 25% or less
- 25% - 50%
- 50% - 75%
- 75% or more

Do you report wolf depredations differently on Public versus Private lands?

- Yes
- No
- I'm not sure

Please explain why you report differently,

(Remember, all responses will remain anonymous).

Please select the type of Public lands where you graze livestock. (select all that apply, all responses will remain anonymous)

- U.S. Forest Service lands (USFS)
- State lands
- Bureau of Land Management lands (BLM)
- ________ Other

TPB Compensation

The following questions will ask for your opinions on wolf depredation compensation. The compensation process is defined as the process of applying for, and receiving compensation for wolf depredations. All responses will remain anonymous.

Have you ever applied for compensation for wolf depredations?

- Yes
- No
- I'm not sure

From which agency or organization do you, (or did you in the past) receive financial assistance for wolf depredation? Please list all agencies and/or organizations if you have received, or currently receive compensation from more than one group.

How satisfied are you with your current compensation program?

- Completely satisfied
- Mostly satisfied
- Somewhat satisfied
- Neither satisfied or dissatisfied
- Somewhat dissatisfied
- Mostly dissatisfied
- Completely dissatisfied

If you've ever chosen NOT to apply for compensation for a wolf depredation, please explain why.

(All responses will remain anonymous).

If you experience wolf depredation in the future, how likely are you to apply for compensation?

- Extremely likely
- Likely
- Neither likely or unlikely
- Unlikely
- Extremely unlikely

Would you say your general attitude towards compensation for wolf depredations is positive, negative, or neutral?

- Extremely positive
- Positive
- Neither positive or negative
- Negative
- Extremely negative

Please indicate to what extent you agree or disagree with the following statements:

The process of applying for wolf depredation compensation is difficult.

- Strongly agree
- Agree
- Somewhat agree
- Neither agree or disagree
- Somewhat disagree
- Disagree
- Strongly disagree

The process of applying for wolf depredation compensation is time consuming.

- Strongly agree
- Agree
- Somewhat agree
- Neither agree or disagree
- Somewhat disagree
- Disagree
- Strongly disagree

Without compensation for wolf depredations, my business would be financially vulnerable.

- Strongly agree
- Agree
- Somewhat agree
- Neither agree or disagree
- Somewhat disagree
- Disagree
- Strongly disagree

The amount of compensation available to me for wolf depredations is representative of my actual losses.

- Strongly agree
- Agree
- Somewhat agree
- Neither agree or disagree
- Somewhat disagree
- Disagree
- Strongly disagree

In addition to compensation for direct losses, (depredations) I believe livestock producers should be compensated for indirect losses, (weight loss, decreased reproductive rates, increased abortion rates, and veterinary bills for injuries caused by wolves).

- Strongly agree
- Agree
- Somewhat agree
- Neither agree or disagree
- Somewhat disagree
- Disagree
- Strongly disagree

My neighbors and/or community would approve of me applying for wolf depredation compensation.

- Strongly agree
- Agree
- Somewhat agree
- Neither agree or disagree
- Somewhat disagree
- Disagree
- Strongly disagree

What percentage of your neighbors and/or community that experience, (or might experience) wolf depredation do you think apply, (or would apply) for compensation for those depredations?

- 25% or less
- 25% - 50%
- 50% - 75%
- 75% or more

Some states include a multiplier for the fair market value price when compensating for wolf depredations. This multiplier operates to account livestock depredated by wolves but never found by the producer. For example, at a multiplier of 3, each located and confirmed wolf depredation will be paid at 3 times the fair market value for one animal.

Do you believe a multiplier would more accurately represent your direct losses to wolves?

- Yes
- No
- I'm not sure

What multiplier do you think would most accurately represent your direct losses?

- 2
- 3
- 4
- 5
- 6
- [ ] 7 or more, (please specify)

Imagine a multiplier was established to cover both direct and indirect losses, (indirect losses consisting of weight loss, injuries, and/or reduced reproductive rates/increased abortion rates among non-depredated animals). What multiplier do you think would most accurately represent your combined direct and indirect losses?

- ☐ 2
- ☐ 3
- ☐ 4
- ☐ 5
- ☐ 6
- ☐ [____] 7 or more, (please specify)
- ☐ I don't believe a multiplier is necessary for direct or indirect losses

Please rank the following groups in order of who you believe should fund conflict prevention tools and strategies for reducing wolf-livestock conflicts, (including depredation compensation). Rank with a "1" the group you believe SHOULD provide the most, if not all of the funding, down to an "8" for the group you believe should NOT be responsible for providing any funding. Please type the numbers into the text boxes to the left of each group.

[____] Hunting licenses

[____] State taxpayer dollars

[____] Federal taxpayer dollars

[____] Private insurance

[____] Recreationists entering nearby Parks

[____] The landowners themselves

[____] Wolf advocates

[____] Other [____]

Please indicate your level of interest in the following:

A compensation program for wolf depredations run by your state wildlife agency

- Extremely interested
- Somewhat interested
- Neither interested or uninterested
- Somewhat uninterested
- Extremely uninterested

A compensation program for wolf depredations run by the federal Fish and Wildlife Agency

- Extremely interested
- Somewhat interested
- Neither interested or uninterested
- Somewhat uninterested
- Extremely uninterested

A compensation program for wolf depredations run by county officials

- Extremely interested
- Somewhat interested
- Neither interested or uninterested
- Somewhat uninterested
- Extremely uninterested

A compensation program for wolf depredations run by an NGO or conservation organization

- Extremely interested
- Somewhat interested
- Neither interested or uninterested
- Somewhat uninterested
- Extremely uninterested

A compensation program for wolf depredations run by local, community-elected volunteers

- Extremely interested
- Somewhat interested
- Neither interested or uninterested
- Somewhat uninterested
- Extremely uninterested

A compensation program for wolf depredations run by the U.S. Department of Agriculture, (USDA)

- Extremely interested
- Somewhat interested
- Neither interested or uninterested
- Somewhat uninterested
- Extremely uninterested

A compensation program for wolf depredations run by your state Department of Agriculture

- Extremely interested
- Somewhat interested
- Neither interested or uninterested
- Somewhat uninterested
- Extremely uninterested

Vignette design

We are interested in getting your perspective on what an ideal producer support program would look like for producers operating on landscapes with depredating wolves. The following question will ask you to choose between five different programs. Each program has three components: a payment option for direct losses, a habitat lease option, and a cost share option. A participant in any of the five programs can take advantage of all, some, or none of the three payment options.

Please review the following details on each payment option, and then select which compensation program you would most prefer to participate in.

Payment Options:

1. Payment for Direct Losses

Similar to traditional depredation compensation programs, livestock depredations that are located, confirmed by the required personnel, and submitted for compensation can receive a fair market value payment per depredated animal. This payment option may or may not include a multiplier for compensation above the fair market value to account for depredated livestock never found. For example, at a multiplier of 3, each located and confirmed wolf depredation will be paid at 3-times the fair market value for one animal.

2. Habitat Lease that Does Not Displace Livestock

An annual payment made to landowners similar to the Conservation Reserve Program (CRP Grasslands), which pays landowners and operators to protect grasslands, including rangelands, pasturelands, and certain other lands while maintaining the areas as grazing lands. This habitat lease option would pay agricultural producers a dollar amount per acre for operating on landscapes with wolves. Participation in the habitat lease would require an initial biodiversity evaluation of lands. Livestock producers operating on both private and/or public lands qualify to participate.

3. Cost Share Program for Wolf Conflict Prevention Tools

A cost-share program for voluntary implementation of proactive conflict prevention and/or non-lethal management tools. As participants, landowners will be provided both financial and technical assistance in the form of equipment and/or services (for example installing fencing). These resources will be paid for by a shared pool of investments funded by NGO's, government agencies, and/or producers. Producer contributions may be in-kind, (labor, materials, etc.) or cash.

Please review the 5 producer support programs below, and then select which of the 5 programs you would most prefer to participate in

**Programs**	**Payment Option 1:**<br><br>Payment for Direct Losses	**Payment Option 2:**<br><br>Habitat Lease that does not displace livestock	**Payment Option 3:**<br><br>Cost Share Program for wolf conflict prevention tools
**1**	Fair Market Value with a multiplier of 3	Not available	Financial assistance and technical assistance provided with cost-sharing
**2**	Fair Market Value	$5 - $9 /acre annually based on geographic location	Financial assistance and technical assistance provided with cost-sharing
**3**	Not available	$10 or more /acre annually based on geographic location	Financial assistance and technical assistance provided with cost-sharing
**4**	Fair Market Value	$10 or more /acre annually based on geographic location	No assistance available
**5**	Fair Market Value with a multiplier of 3	$5 - $9 /acre annually based on geographic location	No assistance available

Which program would you most prefer to participate in?

- Option 1
- Option 2
- Option 3
- Option 4
- Option 5

To what extent would you be satisfied with the program you selected as most preferable?

- Completely satisfied
- Mostly satisfied
- Somewhat satisfied
- Neither satisfied or dissatisfied
- Somewhat dissatisfied
- Mostly dissatisfied
- Completely dissatisfied

Keeping in mind that resources are limited, what could be changed about the program you selected as your most preferred option to make the program even more preferable?

Demographic Info

What is your age?

- 18 or younger
- 19 - 29
- 30 - 49
- 50 - 69
- 70 or older

What is your gender?

- Male
- Female

If you would like to be entered into our raffle for a chance to win free issues of "On Land Magazine" from Western Landowners Alliance, please provide your email address below.

Based on the state of residence you selected, we would like to ask you a few questions about your experience with grizzly bears.

Have you ever experienced a grizzly bear depredation on the livestock you own or manage?

- Yes
- No
- I'm not sure

Was there ever a time when you chose NOT to report a grizzly bear depredation?

- Yes
- No
- I'm not sure

Why didn't you report the grizzly bear depredation(s)? All responses will remain anonymous.

Powered by Qualtrics
