# Supplementary material for: Drivers of wolf depredation reporting and compensation use intentions by livestock producers
Source: PeerJ. 2026 Feb 2;14:e20732. doi: 10.7717/peerj.20732 (PMC12875219; doi:10.7717/peerj.20732)
Supplement: Supplemental Information 7 [file peerj-14-20732-s007.docx]

**Supplemental File S1: Details on statistical analyses methods.**

As a pre-screening test to reduce overfitting our models, we ran Lasso regressions using the glmnet package in R (v4. 1-1; Friedman & Tibshirani., 2010) on each of our four models – the specific reporting and compensation use models, and the mixed reporting and compensation use models. Lasso, or Least Absolute Shrinkage Selector Operator regression is a regularization method for variable selection in linear modeling that uses cross-validation to determine both the number of predictors and the appropriate shrinkage (Tibshirani, 1996; Wan et al., 2014; McNeish, 2015; Niemiec et al., 2016; Mainzer at al., 2022). Because Lasso does not easily accommodate multiple imputation datasets, and because we did not want to lose important perspectives by using complete case analysis, we used mean replacement for missing data before running Lasso variable selection (Wood et al., 2008; van Buuren, 2018). Next, we ran Ordinary Least Squares regressions (OLS) on the Lasso-selected variables for each model allowing us to obtain interpretable p-values and standardized beta coefficients (Wan et al., 2014; McNeish, 2015). We confirmed our concern regarding dealing with our incomplete cases in a way that did not impact our final results when our findings showed that both our missingness indicator for age and missingness indicator for past experience with risk were statistically significant (see Tables 2 and 3), and we would have lost that information by removing those responses before analysis. As a sensitivity test, we ran all four models using multiple imputation via chained equations selecting one imputation to run OLS on the Lasso selected variables (MICE – van Buuren & Groothuis-Oudshoorn, 2025) instead of mean replacement (Wan et al., 2014; van Buuren, 2018; Mainzer et al., 2022). We found very similar results across both methods, validating our mean-replacement approach.

Missingness-indicators created during mean-replacement were not included in multiple imputation and therefore marked with “NA” under the multiple imputation models in Tables 2 and 3. Similarly, predictors found significant by Multiple Imputation but not significant during mean-replacement are marked with “NA” under the mean-replacement models in Tables 2 and 3. Finally, Benjamin-Hochberg adjustments were made to p-values on both the mean-imputed, and multiple imputation regressions to account for multiple testing and control for the false discovery rate (Narum, 2006; Benjamin & Hochberg, 2018). Unlike Bonferroni corrections that can be overly conservative for exploratory studies, the Benjamin-Hochberg procedure balances the risk of Type I error with the need to maintain statistical power, limiting the proportion of false positives and allowing for a more nuanced interpretation (Narum, 2006; Benjamin & Hochberg, 2018).

**References:**

**Benjamini, Y., & Hochberg, Y. (1995).** Controlling the false discovery rate: A practical and powerful approach to multiple testing. Journal of the Royal Statistical Society: Series B (Methodological), 57(1), 289–300. <https://doi.org/10.1111/j.2517-6161.1995.tb02031.x>

**Mainzer, R. M., Nguyen, C. D., Carlin, J. B., Moreno-Betancur, M., White, I. R., & Lee, K. J. (2022).** A comparison of strategies for selecting auxiliary variables for multiple imputation. Statistical Methods in Medical Research, 31(4), 1063–1075. <https://doi.org/10.1177/09622802221102712>

**McNeish, D. M. (2015).** Using Lasso for predictor selection and to assuage overfitting: A method long overlooked in behavioral sciences. Multivariate Behavioral Research, 50(5), 471–484. <https://doi.org/10.1080/00273171.2015.1036965>

**Narum, S. R. (2006).** Beyond Bonferroni: Less conservative analyses for conservation genetics. Conservation Genetics, 7(5), 783–787. <https://doi.org/10.1007/s10592-005-9056-y>

**Niemiec, R. M., Ardoin, N. M., Wharton, C. B., & Asner, G. P. (2016).** Motivating residents to combat invasive species on private lands: Social norms and community reciprocity. Ecology and Society, 21(2), Article 18. <https://www.jstor.org/stable/26270381>

**Tibshirani, R. (1996).** Regression shrinkage and selection via the Lasso. Journal of the Royal Statistical Society: Series B (Methodological), 58(1), 267–288. <https://doi.org/10.1111/j.2517-6161.1996.tb02080.x>

**van Buuren, S. (2018).** Flexible imputation of missing data (2nd ed.). Chapman & Hall/CRC.

**van Buuren, S., & Groothuis-Oudshoorn, K. (2011).** mice: Multivariate imputation by chained equations in R. Journal of Statistical Software, 45(3), 1–67. <https://doi.org/10.18637/jss.v045.i03>

**Wan, Y., Datta, S., & Conklin, D. J. (2015).** Variable selection models based on multiple imputation with an application for predicting median effective dose and maximum effect. Journal of Statistical Computation and Simulation, 85(9), 1803–1815. <https://doi.org/10.1080/00949655.2014.907801>

**Wood, A. M., White, I. R., & Royston, P. (2008).** How should variable selection be performed with multiply imputed data? Statistics in Medicine, 27(17), 3227–3246. <https://doi.org/10.1002/sim.3177>
