# Supplementary material for: Drivers of wolf depredation reporting and compensation use intentions by livestock producers
Source: PeerJ. 2026 Feb 2;14:e20732. doi: 10.7717/peerj.20732 (PMC12875219; doi:10.7717/peerj.20732)
Supplement: Supplemental Information 8 [file peerj-14-20732-s008.docx]

**Survey Codebook:**

| **Variable name** | **Description** | **Type** | **Likert scale** | **Coding** |
| --- | --- | --- | --- | --- |
| Cattle | Cattle | categorical | NA | 1 = cattle  0 = not cattle |
| Sheep | Sheep | categorical | NA | 1 = sheep  0 = not sheep |
| HeadCount | Total head of livestock | ordinal | NA | 1 = 500 head or less  2 = between 500 and 1000 head  3 = between 1000 and 3000 head  4 = 3000 head or more |
| PrivateLand | Operate on private, public, or other lands | categorical | NA | 0 = private land  1 = public land  2 = other |
| State | State of residence | categorical | NA | 1 = Arizona  2 = California  3 = Idaho  4 = Montana  5 = New Mexico  6 = Oregon  7 = Washington  8 = Wyoming  9 = Colorado  10 = Alberta |
| Dep | Past experience with wolf depredation (past experience risk) | categorical | NA | 0 = no  1 = yes  2 = I’m not sure |
| Report | have they ever reported a depredation (reporting use) | categorical | NA | 0 = no  1 = yes  2 = I’m not sure |
| FR | Intention to report wolf depredation in the future (dependent variable) | ordinal | 5-point | 1 = extremely unlikely  2 = unlikely  3 = neither likely or unlikely  4 = likely  5 = extremely likely |
| AttitudeR | Attitude reporting (attitude) | ordinal | 5-point | 1 = extremely negative  2 = negative  3 = neither positive or negative  4 = positive  5 = extremely positive |
| Worry | How worried are they about wolf depredation (perceived probability risk) | ordinal | 5-point | 1 = not at all worried  2 = slightly worried  3 = somewhat worried  4 = moderately worried  5 = extremely worried |
| RRecord | Reporting is important for maintaining an accurate record (reporting personal norm) | ordinal | 7-point | 1 = strongly disagree  2 = disagree  3 = somewhat disagree  4 = neither agree or disagree  5 = somewhat agree  6 = agree  7 = strongly agree |
| RTrust | Trust in the depredation investigation process (trust in management/the process) | ordinal | 7-point | 1 = strongly disagree  2 = disagree  3 = somewhat disagree  4 = neither agree or disagree  5 = somewhat agree  6 = agree  7 = strongly agree |
| RNoFeds | I don’t want the government involved in my operation (trust in the federal government) | ordinal | 7-point | 1 = strongly disagree  2 = disagree  3 = somewhat disagree  4 = neither agree or disagree  5 = somewhat agree  6 = agree  7 = strongly agree |
| RNoState | I don’t want the government involved in my operation (trust in the state government) | ordinal | 7-point | 1 = strongly disagree  2 = disagree  3 = somewhat disagree  4 = neither agree or disagree  5 = somewhat agree  6 = agree  7 = strongly agree |
| RNoEnviro | I don’t want environmental groups involved in my operation (trust in environmental groups) | ordinal | 7-point | 1 = strongly disagree  2 = disagree  3 = somewhat disagree  4 = neither agree or disagree  5 = somewhat agree  6 = agree  7 = strongly agree |
| RTimedetect | Time to detect carcasses (reporting perceived behavioral control) | ordinal | 7-point | 1 = strongly disagree  2 = disagree  3 = somewhat disagree  4 = neither agree or disagree  5 = somewhat agree  6 = agree  7 = strongly agree |
| RTimeConfirm | Depredation confirmation process (reporting perceived behavioral control) | ordinal | 7-point | 1 = strongly disagree  2 = disagree  3 = somewhat disagree  4 = neither agree or disagree  5 = somewhat agree  6 = agree  7 = strongly agree |
| RKnowHow | I know who to report depredation to (reporting perceived behavioral control) | ordinal | 7-point | 1 = strongly disagree  2 = disagree  3 = somewhat disagree  4 = neither agree or disagree  5 = somewhat agree  6 = agree  7 = strongly agree |
| RNorm | My neighbors and/or community would support me reporting (reporting injunctive norm) | ordinal | 7-point | 1 = strongly disagree  2 = disagree  3 = somewhat disagree  4 = neither agree or disagree  5 = somewhat agree  6 = agree  7 = strongly agree |
| RNP | Percentage of neighbors and/or community also reporting (reporting descriptive norm) | ordinal | NA | 1 = 25% or less  2 = 25% - 50%  3 = 50% - 75%  4 = 75% or more |
| RIdentify | Reporting helps agencies identify problem wolves (reporting utility belief) | ordinal | 7-point | 1 = strongly disagree  2 = disagree  3 = somewhat disagree  4 = neither agree or disagree  5 = somewhat agree  6 = agree  7 = strongly agree |
| Comp | Have they ever applied for compensation (compensation use) | categorical | NA | 0 = no  1 = yes  2 = I’m not sure |
| AttitudeC | Attitude compensation (attitude) | ordinal | 5-point | 1 = extremely negative  2 = negative  3 = neither positive or negative  4 = positive  5 = extremely positive |
| CDifficult | Applying for compensation is difficult (compensation perceived behavioral control) | ordinal | 7-point | 1 = strongly disagree  2 = disagree  3 = somewhat disagree  4 = neither agree or disagree  5 = somewhat agree  6 = agree  7 = strongly agree |
| CTime | Applying for compensation is time consuming (compensation perceived behavioral control) | ordinal | 7-point | 1 = strongly disagree  2 = disagree  3 = somewhat disagree  4 = neither agree or disagree  5 = somewhat agree  6 = agree  7 = strongly agree |
| CVulnerable | Without compensation I would be financially vulnerable (perceived risk severity) | ordinal | 7-point | 1 = strongly disagree  2 = disagree  3 = somewhat disagree  4 = neither agree or disagree  5 = somewhat agree  6 = agree  7 = strongly agree |
| CAmount | The amount of compensation represents my losses to wolves (compensation beliefs about usefulness) | ordinal | 7-point | 1 = strongly disagree  2 = disagree  3 = somewhat disagree  4 = neither agree or disagree  5 = somewhat agree  6 = agree  7 = strongly agree |
| CNorm | My neighbors and/or community would support me using compensation (compensation injunctive norm) | ordinal | 7-point | 1 = strongly disagree  2 = disagree  3 = somewhat disagree  4 = neither agree or disagree  5 = somewhat agree  6 = agree  7 = strongly agree |
| CNP | Percentage of neighbors and/or community also applying for compensation (compensation descriptive norm) | ordinal | NA | 1 = 25% or less  2 = 25% - 50%  3 = 50% - 75%  4 = 75% or more |
| Age | Age | categorical | NA | 1 = 18 or younger  2 = 19-29  3 = 30-49  4 = 50-69  5 = 70 or older |
| Gender | Gender | categorical | NA | 1 = male  2 = female |
| FC | Intention to apply for compensation in the future (dependent variable) | ordinal | 5-point | 1 = extremely unlikely  2 = unlikely  3 = neither likely or unlikely  4 = likely  5 = extremely likely |
