# Supplementary material for: Drivers of wolf depredation reporting and compensation use intentions by livestock producers
Source: PeerJ. 2026 Feb 2;14:e20732. doi: 10.7717/peerj.20732 (PMC12875219; doi:10.7717/peerj.20732)
Supplement: Supplemental Information 9 — N = 130 respondents (unless otherwise specified with **) with percentage of the sample for each survey question. [file peerj-14-20732-s009.docx]

**Table S1:**

**Survey constructs, questions used to measure each construct, and descriptive results wolf depredation reporting and compensation use intentions survey.**

N = 130 respondents (unless otherwise specified with **) with percentage of the sample for each survey question.

| **Construct** | **Survey Questions** | **Results – Percent Total Sample** |
| --- | --- | --- |
| **TPB Reporting:**  *Attitude  *Perceived behavioral  control  *Injunctive norm  *Descriptive norm  *Personal norm  **TPB Compensation:**  *Attitude  *Perceived behavioral  control  *Injunctive norm  *Descriptive norm  **Utility Beliefs:**  *Compensation  *Reporting  **Trust:**  *Management/process    *Federal government  *State government  *Environmental groups  **Perceived Risk:**  *Perceived risk severity  *Perceived probability  risk  *Past experience risk | *Would you say your general attitude towards reporting wolf depredations to the required personnel is positive, negative, or neutral?  *I know who to call to report wolf depredations.  *Detecting carcasses depredated by wolves is time consuming.  *Having carcasses confirmed by the required personnel as wolf depredations is time consuming  *My neighbors and/or community would approve of me reporting wolf depredations.  *What percentage of your neighbors and/or community that experience, (or might experience) wolf depredations do you think report, (or would report) those depredation(s)?  *Reporting is important for maintaining an accurate record of wolf depredation.  *Would you say your general attitude towards compensation for wolf depredations is positive, negative, or neutral?  *The process of applying for wolf depredation compensation is difficult.  *The process of applying for wolf depredation compensation is time consuming.  *My neighbors and/or community would approve of me applying for wolf depredation compensation.  *What percentage of your neighbors and/or community that experience, (or might experience) wolf depredations do you think apply, (or would apply) for compensation for those depredation(s)?  *The amount of compensation available to me is representative of my actual losses.  *Reporting wolf depredation helps wildlife management agencies identify depredating wolves.  *I trust the personnel investigating a wolf depredation to investigate fairly.  *I don't want the federal government involved in my operations.  *I don't want the state government involved in my operations.  *I don't want environmental groups involved in my operations.  *Without compensation for wolf depredations, my business would be financially vulnerable  *Typically, how worried are you about wolf depredations on your livestock?  *Have you ever experienced wolf depredation? | *Extremely Positive-Positive*  51%  *Agree-Strongly Agree*  87%  67%  76%  72%  50-100% of my neighbors = 76%  79%  *Extremely Positive-Positive*  45%    *Agree-Strongly Agree*  29%  42%  65%  50-100% of my neighbors = 67%  *Agree-Strongly Agree*  12%  66%  *Agree-Strongly Agree*  44%  37%  31%  61%  *Agree-Strongly Agree*  35%  *Moderately Worried - Extremely Worried*  59%  Yes = 43%  No = 50%  Not sure = 6% |
