# Supplementary material for: Drivers of wolf depredation reporting and compensation use intentions by livestock producers
Source: PeerJ. 2026 Feb 2;14:e20732. doi: 10.7717/peerj.20732 (PMC12875219; doi:10.7717/peerj.20732)
Supplement: Supplemental Information 10 — N = 130 total respondents (unless otherwise specified with **) with percentage of the sample for each survey question. [file peerj-14-20732-s010.docx]

**Table S2:**

**Survey constructs, questions used to measure each construct, and descriptive results wolf depredation reporting and compensation use intentions survey.**

N = 130 total respondents (unless otherwise specified with **) with percentage of the sample for each survey question.

| **Construct** | **Survey Questions** | **Results – Percent Total Sample** |
| --- | --- | --- |
| **Reporting and Compensation:**  *Reporting use  *Compensation use  *Intention to report  (dependent variable)  * Intention to use  compensation  (dependent variable) | *Have you ever reported a wolf depredation?  ** n = 56  *Was there a time when you chose NOT to report a wolf depredation?  ** n = 48  *Have you ever applied for compensation for wolf depredation(s)?  *If you experience wolf depredation in the future, how likely are you to report the depredation(s)?  *If you experience wolf depredation in the future, how likely are you to apply for compensation? | Yes = 86%  No = 14%  Not sure = 0%  Yes = 23%  No = 75%  Not sure = 2%  Yes = 30%  No = 70%  Not sure = 1%    *Likely–Extremely Likely*  80%  79% |
| **Demographics:**  *Location  *Age  *Gender *Type of livestock  *Number of head  *Type of land grazed | Alberta = 5%, Arizona = 3%, California = 15%, Colorado = 14%, Idaho = 3%, Montana = 27%, New Mexico = 9%, Oregon = 6%, Washington = 0%, Wyoming = 14%  0-18 = 0%, 19-29 = 4%, 30-49 = 23%, 50-69 = 53%, 70+ = 21%  Male = 72%, Female = 28%  Cattle = 75%, Sheep = 14%, Goats = 6%, Other = 6%  500 or less = 57%, 500-1000 = 21%, 1000-3000 = 12%, 3000 + = 10%  Private = 57%, Public = 37%, Other = 6% | |
